# Fit for purpose high‐throughput absolute quantitation of chimeric aducanumab in mouse cortex and plasma

**DOI:** 10.1002/alz.093048

**Published:** 2025-01-03

**Authors:** Emma H. Doud, Kathryn A Haynes, Diogo Francisco Silva Santos, Amber L. Mosley, Sara K Quinney, Stacey J Sukoff Rizzo, Paul R Territo

**Affiliations:** ^1^ Indiana University School of Medicine, Indianapolis, IN USA; ^2^ University of Pittsburgh School of Medicine, Pittsburgh, PA USA; ^3^ University of Pittsburgh, Pittsburgh, PA USA; ^4^ Dep. of Biochemistry and Mol. Biology, Indiana Univ. Sch. of Med, Indianapolis, IN USA; ^5^ Indiana University, Indianapolis, IN USA

## Abstract

**Background:**

Although pharmacokinetics and pharmacodynamics of biotherapeutics are commonly studied through ELISAs; however, the extremely strong binding of modern antibody‐based therapeutics result in background, inability of secondary antibody binding, and nonlinear response curves. The selectivity and specificity imparted through the use of liquid chromatography‐targeted mass spectrometry (LC‐MS/MS) allows for absolute quantitation of chosen peptides. For MODEL‐AD, here we present a high‐throughput workflow for absolute quantification of chimeric aducanumab from cortex and plasma of 5XFAD mice.

**Methods:**

A targeted MS assay for quantitation of aducanumab was designed utilizing guidelines described by the National Cancer Institute’s Clinical Proteomic Tumor Analysis Consortium. Proteotryptic peptides unique for chimeric aducanumab were selected, and stable isotope versions were purchased as spike in controls. Given that aducanumab was present in mouse cortex at very low levels, a high sensitivity and high throughput methodology was optimized with Protein A enrichment, reduction, alkylation, trypsin digestion, loading samples onto Evotips using an AssayMap Bravo (Agilent). Evosep LC was paired with a Lumos Tribrid orbitrap (Thermo Fisher Scientific) and data were analyzed in Skyline (MacCoss lab) with a concentration curve of pure protein in matrix normalized to spike in stable isotope labeled peptides.

**Results:**

The three tryptic peptides used for quantitation of aducanumab had lower limits of detection and quantification of 1‐500 Amol pure peptide on column and 2‐5 ng aducanumab/uL in plasma and 0.225 ng/ug brain homogenate. This assay was sensitive and linear over 1 to 500,000 Amol range with high reproducibility (CV 3‐10%). Using a protein A purification, the lower limit of quantification was decreased by 100 fold. This assay was micronized for 96 sample formats, where a single plate could be analyzed in 48‐72 hours.

**Conclusions:**

Although unique peptides will vary, we anticipate this general workflow will allow for quantitation of AD focused biotherapeutics. As part of the open science framework, this methodology will be made available to the broader research community to facilitate broad application.

